# Evaluation of the Remineralisation and Antibacterial Properties of Propolis, Chitosan, and Theobromine

**DOI:** 10.3290/j.ohpd.c_2706

**Published:** 2026-05-28

**Authors:** Elif Yazan Sukur, Yeliz Guven, Nursen Topcuoglu, Sule Batu, Kaan Orhan, Elif Bahar Tuna Ince

**Affiliations:** a Elif Yazan Sukur Assistant Professor, Department of Paediatric Dentistry, Faculty of Dentistry, Istanbul Okan University, Istanbul, 34947, Türkiye; participated in all aspects of the laboratory work, contributed to data acquisition and interpretation, and drafted the manuscript.; b Yeliz Guven Associate Professor, Department of Pedodontics, Faculty of Dentistry, Istanbul University, Fatih 34134, Istanbul, Türkiye; wrote the manuscript and contributed to data analysis.; c Nursen Topcuoglu Professor, Department of Basic Medical Sciences, Faculty of Dentistry, Istanbul University, Fatih 34134, Istanbul, Türkiye; contributed to study conceptualisation and managed the relevant laboratory analyses, proofread the manuscript.; d Sule Batu Assistant Professor, Department of Basic Medical Sciences, Faculty of Dentistry, Istanbul University, Fatih 34134, Istanbul, Türkiye; contributed to study conceptualisation and managed the relevant laboratory analyses; proofread the manuscript.; e Kaan Orhan Professor, Department of Dentomaxillofacial Radiology, Faculty of Dentistry, Ankara University, 06560, Ankara, Türkiye; Medical Design Application and Research Center (MEDITAM), Ankara University, 06560, Ankara, Türkiye; Department of Oral Diagnostics, Faculty of Dentistry, Semmelweis University, 1088, Budapest, Hungary; contributed to study conceptualisation and managed the relevant laboratory analyses; proofread the manuscript.; f Elif Bahar Tuna Ince Professor, Department of Pedodontics, Faculty of Dentistry, Istanbul University, Fatih 34134, Istanbul, Türkiye; conceptualised the study, contributed to the experimental design, and assisted with data interpretation; proofread the manuscript.

**Keywords:** antibacterial, chitosan, propolis, remineralisation, theobromine

## Abstract

**Purpose:**

This study evaluated the remineralisation potential of the natural compounds propolis, chitosan, and theobromine on artificial initial carious lesions and investigated their antibacterial and anti-biofilm activities.

**Methods and Materials:**

Artificial lesions were induced on buccal and lingual enamel surfaces of 14 extracted molars through a 72-h demineralisation process. Specimens were randomly assigned to seven groups (n = 4): propolis (100, 200 μg/mL), chitosan (1.25, 2.5 mg/ml), theobromine (100, 200 mg/L), and artificial saliva (control). Mineral density was assessed using micro-computed tomography (micro-CT) at baseline, after demineralisation, and after pH cycling. The minimum inhibitory concentration (MIC) was determined by broth microdilution, and antibiofilm effects were evaluated using confocal laser scanning microscopy (CLSM).

**Results:**

Micro-CT showed that both propolis groups exhibited mineral gain and % remineralisation values similar to the negative control, whereas chitosan and theobromine groups had significantly higher values (*P* < 0.05). MIC values were 100 μg/ml for propolis and 0.15 mg/ml for chitosan, while theobromine showed no effect. Quantitative CLSM analysis revealed that only the propolis 200 µg/ml group had a significantly lower total biomass compared to the negative control (*P* < 0.05). Additionally, both propolis groups demonstrated a significantly higher dead/live cell ratio than the negative control groups (*P *< 0.05). However, no significant differences in dead/live cell ratios were observed among the chitosan, theobromine, and negative control groups (*P* > 0.05).

**Conclusion:**

Chitosan and theobromine exhibited favourable outcomes in terms of remineralisation; however, their antimicrobial efficacy remained limited. In contrast, propolis exhibited promising antibacterial activity but lacked significant remineralisation capability.

Dental caries has been described as a non-communicable disease primarily caused by a dysbiosis in the dental biofilm, representing a disruption in the balance of the oral microbiome.^25,44^ The presence of sucrose and other fermentable carbohydrates, particularly simple sugars such as monosaccharides and disaccharides, shifts the oral microbiome from a healthy state (symbiosis) to an imbalanced, disease-promoting state (ecological dysbiosis). Cariogenic biofilms generate high levels of acids that are released into the surrounding dental biofilm fluid, disrupting the mineral balance in enamel and dentin. This imbalance leads to a breakdown in the biofilm homeostasis, causing a shift from mineral gain to mineral loss.^25,51^ When the rate of demineralisation surpasses that of remineralisation in the subsurface layers of a tooth, mineral dissolution begins, leading to the formation of clinically visible white spot lesions, which is an initial stage of cavities. White spot lesions are reversible caries lesions when preventive protocols are established; otherwise, subsurface demineralisation progresses, ultimately resulting in surface disintegration and cavity formation.^33^

Noninvasive treatment of noncavitated carious lesions through remineralisation restores both the structural integrity and aesthetic properties of enamel and is a key focus of modern dentistry. Topical fluoride, widely regarded as a highly effective agent in evidence-based caries prevention, reduces mineral loss in dental tissues and promotes remineralisation.^10^ Despite its proven effectiveness in preventing caries, concerns regarding the side effects of systemic fluoride use, along with the FDA’s assertion that fluoride deficiency neither causes disease nor disrupts homeostasis, have raised questions about the necessity of systemic fluoride intake.^22^ These concerns have led to increased interest in alternative remineralisation agents with antibacterial properties. Bioactive materials, calcium- and phosphate-based remineralisation agents, and herbal compounds are emerging as viable fluoride-free options for promoting both remineralisation and antibacterial effects.^4,6,29^

Propolis is a natural substance produced by honeybees from resinous exudates collected from tree and plant buds. These exudates are enzymatically processed and combined with beeswax to create a bioactive compound used primarily for hive repair and disinfection.^8^ Its phenolic components contribute to its antibacterial properties by inhibiting the adhesion and colonisation of microorganisms, altering cell membrane permeability, and reducing adenosine triphosphate (ATP) production within the cell membrane.38 Research indicates that ethanolic extracts of propolis, with effects varying based on the solvent used, exhibit antimicrobial activity against cariogenic bacteria such as *S. mutans* and also play a role in inhibiting demineralisation.^16,34,35,38^

Chitosan, another promising anticariogenic agent, is a non-toxic polymer derived from the deacetylation of chitin, which is found in the exoskeletons of arthropods and certain marine organisms.^21^ Its positive ion charge allows it to bind to bacterial cell walls, conferring both bactericidal and bacteriostatic effects.^27^ Similar to propolis, chitosan is recognised for its antibacterial, antiviral, anti-inflammatory, antioxidant, and antifungal properties.^1^ A recent meta-analysis reported that chitosan-containing chewing gum has an inhibitory effect on *S. mutans*.^42^ Chitosan-containing toothpaste has been shown to achieve similar results in surface hardness compared to fluoride-based toothpaste.^12^ Furthermore, studies indicate that chitosan may support remineralisation in white spot lesions by inhibiting demineralisation.^5,6,26^

Theobromine (3,7-dimethylxanthine), a natural compound found in cocoa bean shells and certain tea plants, is another agent recognised for its caries-preventing effect. An isomer of caffeine and theophylline, theobromine is soluble in water and alcohol and has demonstrated anti-inflammatory and antitumour effects.^24^ Recent studies indicate that theobromine, in the presence of calcium and phosphate, promotes the formation of larger hydroxyapatite crystals rather than simply stimulating mineral precipitation on the enamel surface. These larger hydroxyapatite crystals exhibit greater resistance to acid attacks compared to normally sized hydroxyapatite molecules.^3,45^ Additionally, theobromine has shown antibacterial activity against *S. mutans* by inhibiting biofilm formation, likely through the action of its flavonoids and phenolic compounds.^40^

Although the remineralisation and antibacterial effects of propolis, chitosan, and theobromine have been studied individually, a comprehensive comparison of these three natural substances is notably lacking in the literature. This study aimed to assess the remineralisation potential of propolis, chitosan, and theobromine on artificial caries lesions using micro-computed tomography (micro-CT). Additionally, their antibacterial effects were evaluated against planktonic *S. mutans* through minimum inhibitory concentration (MIC) and minimum bactericidal concentration (MBC) assays, and their impact on *S. mutans* biofilms was analysed using confocal laser scanning microscopy (CLSM). The null hypotheses were that: (I) the tested natural compounds have no effect on the remineralisation of artificial caries lesions, (II) the tested natural compounds have no antibacterial efficacy against S. mutans.

## MATERIALS AND METHODS

Ethical approval was obtained from the Ethics Committee of Istanbul University Faculty of Dentistry (reference no: 2019/61). All participants were informed before extraction that their teeth would be used for research purposes, and written informed consent was obtained before the experiment.

Sample size calculation was performed using G*Power software.^23^ For the antibacterial activity test, the parameters were set at a 5% significance level, 80% power, and a large effect size (d = 4.850), based on data from a previous study,^18^ resulting in a required sample size of 5 per group. For the remineralisation experiment, a separate calculation was conducted using a 5% significance level, 95% power, and a large effect size (d = 1.076), according to another published study,^11^ which indicated a required sample size of four specimens per group.

### Evaluation of Remineralisation Potential

#### Specimen preparation

Extracted human third molars, which had not been previously treated and were free of caries, cracks, or developmental defects, were collected and cleaned of any soft tissue remnants. They were stored in 0.1% thymol solution at 4°C and used within 3 months after extraction. The teeth were then embedded in self-curing acrylic resin (Imicryl Acrylic Repair Material, Konya, Türkiye) blocks using circular moulds of 2 cm in diameter and 2 cm in depth, keeping the roots within the mould. Enamel surfaces were covered with nail varnish (Flormar, Italy), leaving a working window on both buccal and lingual surfaces using adhesive tape (approximately 5mm wide and 5mm long). After allowing the nail varnish to dry for 24 h, the adhesive tape was removed, and any tape residues were cleaned with cotton pads soaked in ethyl alcohol. Once the samples were prepared, baseline micro-CT evaluations (Skyscan 1172; Bruker, Kontich, Belgium) were conducted for mineral density (MD) calculations (baseline MD = T0).

#### Incipient carious lesion creation

To induce initial caries lesions, the specimens were immersed in a demineralisation solution consisting of distilled water, 2.0 mM Ca(NO_3_)_2_, 2.0 mM Na_2_HPO_4_, and 75 mM acetic acid at pH 4.3 for 72 h.^5^ The solutions containing the specimens (10 ml per sample) were refreshed every 24 h. Upon completion of the demineralisation process, the specimens were rinsed with deionised water to eliminate any excess solution, and the mineral densities of the demineralised surfaces were determined using micro-CT (demineralisation MD = T1).

#### Experimental groups

The specimens were randomly divided into seven groups, with each group consisting of 2 teeth and 4 surfaces (buccal and lingual), resulting in a sample size of 4 per group (n = 4). The groups and the properties of the test solutions are summarised in Table 1.

**Table 1:** Experimental groups and properties of the test solutions

Group	Solution applied	Concentration	pH	Source
1	Propolis	100 μg/ml (0.01%)	6.2	Altiparmak, Istanbul, Türkiye
2	Propolis	200 μg/ml (0.02%)	6.2	Altiparmak, Istanbul, Türkiye
3	Chitosan	1.25 mg/ml	6.0	Sigma-Aldrich (product no: 448869), USA
4	Chitosan	2.5 mg/ml	6.0	Sigma-Aldrich (product no: 448869), USA
5	Theobromine	100 mg/L	6.0	Cayman Chemical (product no: 21745), USA
6	Theobromine	200 mg/L	6.0	Cayman Chemical (product no: 21745), USA
7	Artificial saliva (negative control)	1.5 mM CaCl_2_, 0.9 mM NaH_2_PO_4_, 0.15 M KCl	7.0	Buzalaf et al^10^


### Preparation of Solutions

#### Artificial saliva

Artificial saliva for the thermodynamic pH cycle was prepared by mixing 1.5 mM CaCl_2_, 0.9 mM NaH_2_PO_4_, and 0.15 M KCl, with the pH adjusted to 7 using 0.15 M KOH, as described by Buzalaf et al.^10^

#### Propolis

Brown Anatolian propolis, identified as ‘Türkiye 3’ in the study by Seyhan et al, was kindly provided by Dr Mehmet Fatih Seyhan, who previously characterised the sample using HPLC analysis.^43^ The raw propolis was originally obtained from Altiparmak (Istanbul, Türkiye). A stock solution was prepared by dissolving 1 g of propolis in 100 ml of 70% ethanol. The mixture was kept in a shaker at room temperature for 24 h, the flask was wrapped with aluminium foil to prevent light exposure. After this period, the solution was filtered through a 0.45 μm membrane filter. The filtrate was then diluted with a 1:3 (v/v) mixture of dimethyl sulfoxide (DMSO) and artificial saliva to prepare working solutions at concentrations of 100 μg/ml (0.01%) and 200 μg/ml (0.02%). The final pH of the working solutions was 6.2.

#### Chitosan

Chitosan (product no: 448869, Sigma-Aldrich, USA) in amounts of 0.625 g and 1.25 g was each dissolved in 100 ml of 1% (v/v) acetic acid at room temperature using a magnetic stirrer until a fully transparent solution was obtained. Working solutions with final chitosan concentrations of 1.25 mg/ml and 2.5 mg/ml were then prepared by mixing the chitosan stock solutions with DMSO (D8418, Sigma-Aldrich, USA) and artificial saliva in a 4:1:15 (v/v/v) ratio, and the final pH was adjusted to 6.0 using 0.1 N NaOH.

#### Theobromine

A stock solution was prepared by dissolving 1 g of theobromine (product no: 21745, Cayman Chemical, Michigan, USA) in 250 ml of DMSO (D8418, Sigma-Aldrich, USA). Working solutions at concentrations of 100 mg/L and 200 mg/L were then prepared by diluting the stock solution with a 1:3 (v/v) mixture of DMSO and artificial saliva. The final pH was adjusted to 6.0 using 0.1 N NaOH.

#### pH cycle and micro-CT analysis

To replicate the oral environment, the remineralisation process was carried out using a 7-day pH cycling model.^46^ Each daily cycle consisted of 6 h of demineralisation, followed by a 2-min treatment with the test medium, 18 h of exposure to an artificial saliva solution, and a second 2-min treatment with the test medium. Each specimen underwent a remineralisation step with 10 ml of the test solution. Between treatments, the specimens were rinsed with distilled water for 5 s. Throughout the demineralisation and remineralisation phases, samples were maintained at 37°C. After the 7-day period, the samples were rinsed again with distilled water for 5 s and allowed to dry. MD was then evaluated using micro-CT after the pH cycling process to assess remineralisation, referred to as remineralisation MD (T2).

Micro-CT was used to assess changes in MD (g/cm^3^) at three time points (T0, T1, and T2) in the test areas. Scans were conducted following standard procedures and in the same position. To ensure the radiographic beam was perpendicular to the buccal and lingual surfaces, the specimens were secured to a computer-controlled turntable. Digital sectional images were acquired under the following conditions: 100 kV voltage, 104 mA current, 0.5 aluminium filter, 1000 × 1000 resolution dpi, and a 360° rotation at a 0.75° step. Following the scanning process, 1200 RAW images in DICOM format were converted to BMP and restructured using NRecon (Ver 1.7.1, Skyscan, Kontich, Belgium). Each cross-sectional image had a resolution of 1000 × 1000 pixels, with a pixel size of 20.00 μm. The raw radiographic images obtained from the micro-CT scan underwent processing in the NRecon 1.7.1 software (SkyScan, Kontich, Belgium), involving 2 units of image smoothing, 10 units of ring artefact correction, and radiographic hardening. A 70% correction rate was applied to remove image contamination and radiographic artefacts, preparing the image for subsequent MD calculations.

The BMP images were then analysed using computer tomography analysis (CTAn) software (version 1.16.4.2 Skycan, Kontich, Belgium) provided by the manufacturer. After selecting the region of interest (ROI), mineral densities in these areas were calculated using the CaHA calibration (phantom) bar, set at 0.25 and 0.75 g/mm^3^.

The MD values at T0, T1, and T2 for each specimen were used to compute the relative mineral gain due to the application of respective test solutions. Additionally, the remineralisation percentage (%R) was calculated to evaluate the potential of each solution to remineralise artificial initial caries-like lesions. Mineral gain and %R for each specimen were determined using the following formula, as outlined in previous studies.^7,13,50^ In this formula, Δzd represents the MD difference between T0 and T1, while Δzr represents the MD difference between T0 and T2.


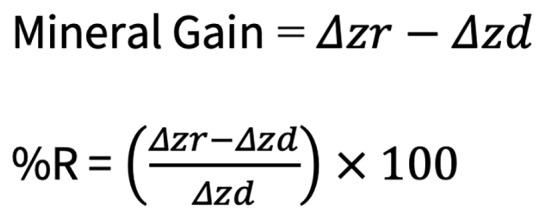


### Antimicrobial Effect Evaluations

#### Bacterial inoculum preparation

A 10 ml culture of *Streptococcus mutans* ATCC 25175, grown for 24 h, was inoculated into 5% sucrose brain heart infusion (BHI) broth to achieve a 0.5 McFarland turbidity standard, corresponding to a concentration of 10⁸ CFU/ml. The culture was then diluted 100-fold to reach a final concentration of 106 CFU/ml. As a control, the suspension was further diluted 10-fold, and 0.1 ml of the final dilution was plated onto solid medium. The inoculated plates were then incubated at 37°C in a CO_2_-enriched environment for 24 h.

#### Determination of minimum inhibitory concentration (MIC) and minimum bactericidal concentration (MBC)

The minimum inhibitory concentration (MIC) and minimum bactericidal concentration (MBC) tests were conducted to evaluate the antimicrobial activity of propolis (200 µg/ml), chitosan (2.5 mg/ml) and theobromine (200 mg/L) solutions, following the reference protocol of the Clinical and Laboratory Standards Institute.^15^ A 2% chlorhexidine solution (Drogsan, Istanbul, Türkiye) was used as the positive control, while artificial saliva +DMSO served as the negative control. MIC testing was performed using 96 well microtiter plates, with each well containing 100 μL of BHI broth. In the first well, 200 μL of the test substance was added, and serial two-fold dilutions (ranging from 1.0 to 0.00048) were performed across the plate, excluding the last well. Subsequently,100 µl of bacterial suspension was added to each well, and the plates were incubated at 37ºC in a 5–7% CO_2_ environment for 24 h. The MIC was determined as the lowest concentration at which no visible bacterial growth was observed. To determine the MBC, 10 µl of the solution was taken from the wells showing no bacterial growth at concentrations higher than the MIC and inoculated on BHI agar plates. The MBC was defined as the lowest concentration at which no colony formation was observed after incubation at 37ºC for 24 h.

MBC/MIC ratio was then calculated to assess the microbicidal effect, with a ratio of ≥4 indicating a bacteriostatic effect and a ratio of < 4 considered bactericidal. To ensure accuracy and reproducibility, all experiments were repeated three times.

#### Biofilm formation

Unstimulated saliva (200 ml), collected from a healthy adult without chronic disease, was centrifuged at 15,000 g at 4°C for 15 min. The resulting supernatant was sterilised using a 0.45 µm filter, and sterility was confirmed by incubating the filtrate on blood agar. The sterile saliva was then stored at –20°C until its use for biofilm tests.

Fifty-four sterile synthetic hydroxyapatite (HA) discs (3D Biotek, USA) were used as substrates to simulate enamel surfaces. HA discs were placed in sterilised 24 well plates containing 500 μl sterile saliva and incubated at 37°C for an hour on a shaker. The discs were rinsed with 2 ml of sterile saline and transferred to a new 24 well plate, where they were inoculated with 200 µl of bacterial suspension and 1.6 ml of BHI supplemented with 5% sucrose. The plates were incubated at 37ºC on a shaker for 24 h to allow biofilm formation. After rinsing with 2 ml of sterile saline, the HA discs were randomly divided into experimental groups. For the antibacterial analysis, the same seven groups used in the remineralisation study were included, along with the addition of phosphate-buffered saline (PBS) as a second negative control, resulting in a total of eight groups (n = 6 per group).

In each well, 750 µl of the test solutions was added to cover the surface of the discs. The HA discs with test solutions were kept on a shaker at 37°C for 4 min. After 4 min, the discs were rinsed in 24 well plates containing PBS for 10 s, repeating the rinse 3 times. The HA discs were transferred to a new sterile 24 well plate. Five samples from each group were allocated for CLSM analysis.

#### Confocal laser scanning microscopy (CLSM) analysis

To quantify live and dead cells, biofilms formed on HA discs were stained using the LIVE/DEAD® BacLightTM Bacterial Viability and Counting Kit (Invitrogen, Thermo Fisher Scientific, USA), which includes two fluorescent nucleic acid stains: SYTO 9 (green), which stains live cells, and propidium iodide (red), which selectively stains dead cells. The staining procedure followed the manufacturer’s instructions. A mixture of 0.8 µl of each stain in 2 ml of DMSO was prepared, and 20 µl of this solution was added to each well, ensuring full coverage of the biofilm on the HA discs. The plates were then covered with aluminium foil and incubated in the dark for 15 min. After staining, the HA discs were examined using a CLSM (Leica TCS SPE; Leica Microsystems, Heidelberg, Germany) at 4× magnification. Specific filters were used with excitation wavelengths of 488 nm for SYTO 9 detection and 543 nm for propidium iodide detection. For each sample, three random fields were selected for scanning. The biomass, defined as the total volume of live and/or dead cells (µm^3^) per unit area of the substratum (hydroxyapatite surface) (µm^2^), was quantified using COMSTAT 2.1 image-processing software. Live and dead biomass were further differentiated and quantified based on the respective staining.

### Statistical Analysis

The data analysis was conducted using IBM SPSS Statistics for Windows, version 22 (IBM, Armonk, NY, USA). Data normality was evaluated using the Kolmogorov–Smirnov and Shapiro–Wilk tests. For data following a normal distribution, one-way analysis of variance (ANOVA) was performed, followed by Tukey’s post hoc test for multiple comparisons. For non-normally distributed data, the Kruskal‒Wallis test was used to compare groups of three or more, with Dunn’s test applied for post hoc analysis. Intragroup comparisons across time points were performed using the Friedman test, followed by the Wilcoxon test for post hoc pairwise analysis. Data visualisation was carried out using GraphPad Prism 10 (GraphPad Prism, San Diego, CA, USA). A significance level of *P* < 0.05 was applied to all statistical tests.

## RESULTS

### Micro-CT Analysis

The MD of the demineralised surface in all groups showed a significant reduction compared to baseline values (*P* ≤.05). After the respective treatments were applied, the MD significantly increased in all groups except for the propolis 100 µg/ml and artificial saliva (negative control) groups, which showed no significant increase (Table 2).

**Table 2:** MD values of the groups at T0, T1, and T2 timepoints

Groups	Mineral densities (g/cm^3^)			Test statistics	P*
	T0	T1	T2		
	Median (min–max)	Median (min–max)	Median (min–max)		
Propolis 100 µg /ml	2.87 (2.83–2.96)a	2.38 (2.36–2.44)b	2.39 (2.33–2.46)b	6	0.05
Propolis 200 µg /ml	2.74 (2.69–2.80)a	2.27 (2.18–2.37)b	2.38 (2.28–2.50)c	8	0.018
Chitosan 1.25 mg/ml	2.83 (2.82–2.95)a	2.37 (2.32–2.41)b	2.79 (2.68–2.89)a	6.5	0.039
Chitosan 2.5 mg/ml	2.85 (2.84–2.97)a	2.30 (2.28–2.33)b	2.80 (2.78–2.83)c	8	0.018
Theobromine 100 mg/L	2.68 (2.64–2.82)a	2.18 (2.15–2.2)b	2.52 (2.42–2.55)c	8	0.018
Theobromine 200 mg/L	2.72 (2.71–2.74)a	2.21 (2.20–2.30)b	2.50 (2.47–2.52)c	8	0.018
Artificial saliva (C–)	2.80 (2.78–2.81)a	2.35 (2.33–2.37)b	2.34 (2.34–2.40)b	6	0.05
*Friedman test, followed by post-hoc Wilcoxon signed-rank test. Different lowercase letters indicate significant differences within each group across T0, T1 and T2 time points (*P* < 0.05). T0 = Baseline, T1 = Demineralisation, T2 = Remineralisation, C– = Negative control					

**Fig 1a and b:**
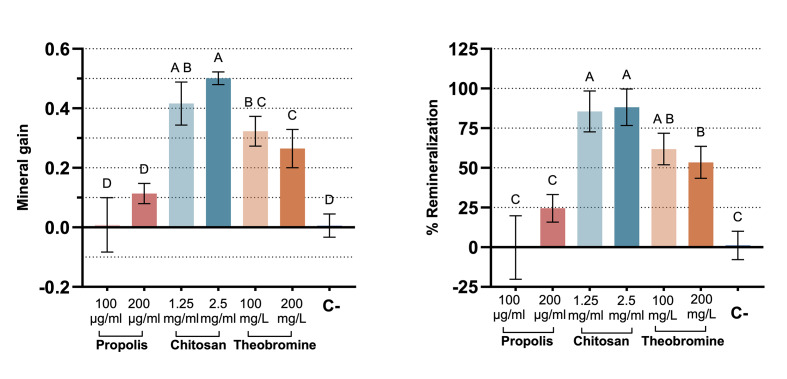
Mineral gain (a) and % remineralisation (b) of each group. Different uppercase letters indicate significant differences among groups (C = Negative control group, artificial saliva).

Figure 1 presents the mineral gain and remineralisation percentage (%R) of each group. Both propolis groups exhibited similar mineral gain and remineralisation potential (%R) to the negative control group, with all showing significantly lower values compared to all chitosan and theobromine groups (*P* < 0.05). Although the chitosan groups demonstrated a higher %R than both the theobromine 100 mg/L and 200 mg/L groups, this difference was statistically significant only when compared to the theobromine 200 mg/L group (*P* < 0.05). In terms of mineral gain, the chitosan 2.5 mg/ml group achieved significantly higher values than both theobromine groups (*P* = 0.078; *P* = 0.01). The chitosan 1.25 mg/ml group showed similar mineral gain to the theobromine 100 mg/L group (*P* = 0.14), but significantly higher values compared to the theobromine 200 mg/L group (*P* = 0.019).

### Antimicrobial Effect Evaluations

Table 3 presents the MIC and MBC values for the experimental and control solutions tested against *S. mutans*. The MIC of propolis was 100 μg/mL, while that of chitosan was 0.15 mg/ml. Theobromine showed no bacteriostatic or bactericidal effects on *S. mutans*. Similarly, the artificial saliva + DMSO solution, used as a cosolvent, exhibited no antibacterial activity. In contrast, chlorhexidine digluconate (%0.2) displayed a strong bacteriostatic effect, with an MIC of 0.001 mg/ml and an MBC of 0.004 mg/ml, highlighting its potent antimicrobial properties even at very low concentrations.

**Table 3:** MIC and MBC of the tested natural compounds on *S. mutans*. The initial stock concentration of each solution is shown, and MIC/MBC values are presented as a dilution ratio relative to the initial concentration

Groups	Initial concentration	MIC (dilution ratio)	MIC (actual value)	MBC (dilution ratio)	MBC (actual value)
Propolis	200 µg/ml	0.5×	100 µg/ml	–	–
Chitosan	2.5 mg/ml	0.0625×	0.15 mg/ml	–	–
Theobromine	200 mg/L	–	–	–	–
Chlorhexidine digluconate	2 mg/ml	0.0005×	0.001 mg/ml	0.002×	0.004 mg/ml
Artificial saliva + DMSO	–	–	–	–	–
* Bactericidal effect = MBC/MIC < 4; bacteriostatic effect = MBC/MIC ≥ 4.					

**Fig 2a to d:**
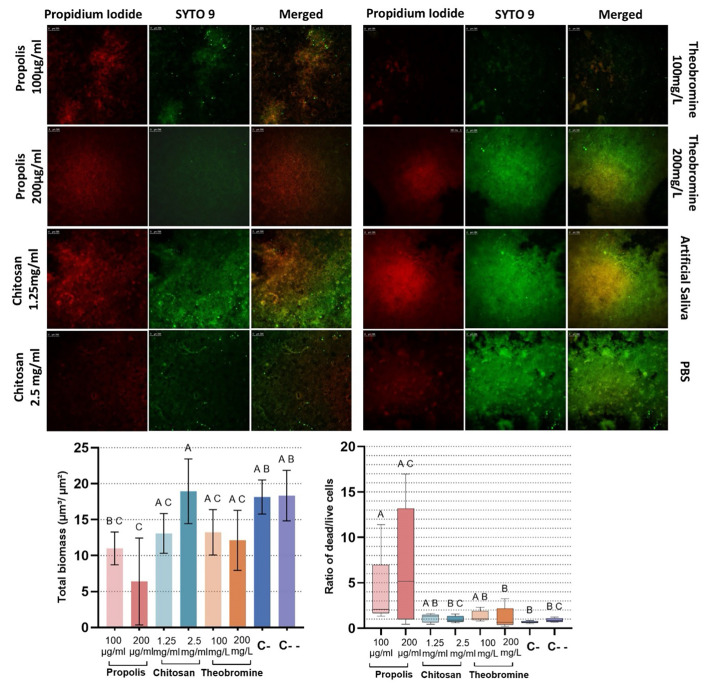
(a and b) Representative confocal microscopy images of biofilm samples from each group. The columns represent the different staining conditions; SYTO 9 stains live cells and propidium iodide stains dead cells, and the merged image shows the overlapping staining pattern (Syto 9 and propidium iodide). The rows represent the different treatments applied (the scale bar is 250 μm). (c) Total biomass (live + dead cells) within the *S. mutans* biofilm on HA discs for each group (*P* < 0.05; one-way ANOVA and post hoc Tukey test) (d) Dead/live cell ratio of each group (*P* ≤ 0.05; Kruskal–Wallis and post hoc Dunn’s test). Different uppercase letters indicate significant differences among groups (C = Negative control group; C– = Artificial saliva, C– = PBS).

The effects of propolis, chitosan, and theobromine solutions on the total biomass and dead/live bacterial ratio within *S. mutans* biofilms on HA discs were analysed using CLSM (Fig 2c and d). Quantitative analysis revealed that, compared to the negative control groups, only the propolis 200 µg/ml group demonstrated a significantly lower total biomass (*P* < 0.05). Additionally, the propolis 100 µg/ml group displayed a significantly higher dead/live cell ratio than chitosan 2.5 mg/ml, theobromine 200 mg/L, and the negative control groups (*P* < 0.05). Similarly, the propolis 200 µg/ml group showed a significantly higher dead/live cell ratio when compared to both the theobromine 200 mg/L and artificial saliva groups (*P* < 0.05). No significant differences in dead/live cell ratios were observed among the chitosan, theobromine, and negative control groups (*P* > 0.05). The CLSM images of *S. mutans* biofilms after each treatment confirmed the quantitative analysis (Fig 2a and b).

## DISCUSSION

The present study aimed to investigate the remineralisation potential and antimicrobial activity of propolis, chitosan, and theobromine solutions at two different concentrations. The remineralisation capacity of the solutions was evaluated by measuring changes in MD at three time points using micro-CT. The antimicrobial activity was tested against *S. mutans* using CLSM analysis. The results indicated that chitosan and theobromine solutions exhibited significantly higher enamel remineralisation potential compared to the negative control, whereas propolis solutions demonstrated results similar to the negative control. Based on these findings, the first null hypothesis of this study was rejected.

Remineralisation in previous studies has been assessed using a scanning electron microscope (SEM) with energy dispersive X-ray spectroscopy (SEM-EDX), microhardness testing, transverse microradiography (TMR), and micro-CT.^3,36,39,47^ However, due to the curved structure of the tooth, SEM-EDX and microhardness testing often require extensive pre-treatment, including the removal of at least 1 mm of enamel, while TMR necessitates the preparation of extremely thin specimens and does not allow for repeated measurements on the same sample over time.^28^ To address these limitations, this study utilised micro-CT, a non-invasive and non-destructive technique that eliminates the need for enamel surface pre-treatment, thereby preserving the integrity of experimental areas while measuring mineral recovery. Furthermore, micro-CT provides comprehensive two- and three-dimensional analyses of MD, surface area, and volume changes, making it a reliable tool for evaluating remineralisation processes.^14^

Cardoso et al investigated the effect of Brazilian wild green propolis on enamel mineral loss using a Knoop microhardness tester. Their results showed that enamel hardness in all groups treated with propolis was comparable to that of the negative control groups (Milli-Q water and 80% ethanol), suggesting that propolis had no significant effect on preventing mineral loss.^11^ Similarly, in the present study, the propolis groups exhibited the lowest remineralisation percentage and mineral gain among all tested natural agents, with results comparable to the negative control group. Cardoso et al attributed the limited effect of Brazilian wild green propolis on mineral loss to its low polyphenol content, which likely reduced its ability to inhibit enamel demineralisation. The propolis used in our study was Anatolian propolis, which is reported to have a high total phenolic content.^30^ Although HPLC analysis was not conducted within the present study, it has been previously performed on the same propolis sample – identified as ‘Türkiye 3’ in the study by Seyhan et al, which identified over 20 phenolic and flavonoid compounds, including caffeic acid, galangin, pinocembrin, chrysin, and quercetin.^43^ Compared to the 33% Brazilian wild propolis used in Cardoso et al’s study, the Anatolian propolis used here had a greater polyphenol variety. However, the limited effect of Anatolian propolis on enamel remineralisation in this study can likely be explained by the low concentrations used (0.01% and 0.02%). In contrast to the findings of this study and those of Cardoso et al, Martins et al reported that a 3% red propolis hydro-alcoholic extract exhibited surface hardness values comparable to those of 0.12% CHX and 0.05% NaF treatment groups, with all three showing significantly less enamel hardness loss than the negative control group.^11,35^ These findings emphasise the importance of both concentration and composition in determining the effectiveness of propolis in preventing enamel mineral loss.

Chitosan has been shown to penetrate up to the dentin–enamel junction and effectively inhibit mineral loss by 55–81%, depending on the concentration and application time used. Arnaud et al identified the optimal conditions as 2.50 mg/ml and 60 s of application.^5^ In this study, chitosan solutions at 1.25 mg/ml and 2.50 mg/ml were applied for 2 min. Unlike the acidic pH of the chitosan solutions used on sound enamel in Arnaud et al’s study, the solutions in this study had a neutral pH and were applied to initial carious lesions. To ensure sufficient interaction with the lesions, the application time was extended to 2 min. Chitosan at both concentrations demonstrated the highest remineralisation efficacy among all groups. These findings align with those of Zhang et al, who reported enhanced surface and subsurface remineralisation in initial carious lesions pretreated with 2.50 mg/ml chitosan before the application of bioactive glass or bioactive glass/polyacrylic acid complexes. Their study, using Knoop microhardness testing, showed increased enamel hardness in chitosan-treated groups, and Raman intensity mapping confirmed a rise in mineral content at both surface and subsurface levels, highlighting chitosan’s role in facilitating effective remineralisation.^52^

Theobromine has been shown to strengthen hydroxyapatite crystals by promoting their growth and altering their structural characteristics, including increased crystal size and enhanced crystallinity. Therefore, these structural changes in the crystal lattice, rather than the mineral content of the enamel, play a key role in improving its resistance to acid challenges.^3^ In the present study, both theobromine groups (100 mg/L and 200 mg/L) exhibited significant remineralisation potential compared to the artificial saliva group. These findings align with a recent systematic review by Silva et al, which identified theobromine as a promising alternative to fluoride for the remineralisation of white spot lesions.45 However, Thorn et al. reported no significant effect of theobromine on demineralisation or remineralisation. In their study, theobromine was incorporated into simulated plaque fluid containing 0.2–1 ppm fluoride, with no fluoride-free control group included. This methodological difference may explain the lack of observed differences between their groups.^47^ In contrast, our study evaluated remineralisation relative to a fluoride-free negative control, allowing the effects of theobromine to be assessed independently of fluoride.

In this study, the antibacterial effects of propolis, chitosan, and theobromine solutions against *S. mutans* were evaluated by determining their MIC and MBC values using the microdilution method. Additionally, their effects on *S. mutans* biofilm were analysed through CLSM. The MBC/MIC ratios indicated that propolis and chitosan solutions exhibited bacteriostatic effects, while the theobromine solution showed no significant antibacterial activity against *S. mutans*. Furthermore, CLSM analysis revealed that the propolis groups had significantly higher dead/live cell ratios compared to all other groups. These findings led to the rejection of the second null hypothesis of this study.

The microdilution method revealed that the theobromine solution did not exhibit antibacterial activity against *S. mutans*. In contrast, propolis and chitosan solutions demonstrated antimicrobial activity, with MIC values of 100 μg/ml and 0.15 mg/ml, respectively. However, their MBC values could not be determined, suggesting that neither solution exhibited bactericidal effects at the tested concentrations. Consistent with our findings, the antibacterial activity of propolis against* S. mutans *has been well-documented in numerous studies.^2,20,31,35,48^ Notably, MIC values for ethanolic extracts of propolis against *S. mutans* vary widely in the literature, ranging from 4 to 625 μg/mL, as reported in the systematic review by Otreba et al.^38^ The variability in antibacterial efficacy across studies reflects differences in propolis composition influenced by regional flora, extraction solvents, and methods. Propolis exhibits antimicrobial activity through the synergistic effects of multiple compounds, including flavonoids (quercetin, pinocembrin, and galangin) and phenolic acids (caffeic and benzoic acid).^38,48^ The Anatolian propolis used in this study, as confirmed by a previous study, was rich in chrysin, galangin, pinostrobin, pinocembrin, and caffeic acid, consistent with the high-quality phenolic composition typical of Anatolian propolis.

In a study by Veloz et al,^49^ conducted to evaluate alternative methods to assess the effects of Chilean propolis on biofilm formation and metabolic activity of *S. mutans*, biofilm formation was assessed using crystal violet staining and CLSM, while metabolic activity was evaluated with MTT and flow cytometry. The results demonstrated that propolis significantly reduced both biofilm formation and metabolic activity in *S. mutans*. However, the authors highlighted that crystal violet staining could yield inaccurate results in differentiating between live and dead bacteria, and they recommended CLSM as a more reliable method for analysing *S. mutans* biofilm structure. In alignment with this recommendation, the present study utilised CLSM to evaluate the *S. mutans* biofilm, revealing that propolis exhibited significantly high antibacterial activity.

In a study by Costa et al, which investigated the antimicrobial effects of chitosan on six oral anaerobic bacteria, including *S. mutans*, the MIC and MBC values for low molecular weight chitosan were reported as 5 mg/ml and 7 mg/ml, respectively.^17^ In the present study, the chitosan used was also of low molecular weight and exhibited an MIC of 0.15 mg/ml against *S. mutans*, with no detectable MBC, indicating a bacteriostatic rather than a bactericidal effect at the tested concentrations. The lower MIC value observed in our study may be attributed to the formulation of chitosan solutions with DMSO and artificial saliva. DMSO, as a strong organic solvent that increases cell membrane permeability, may have facilitated the penetration of chitosan into bacterial cell walls, enhancing its antibacterial activity.^37^ Additionally, differences in *S. mutans* strains used and variations in experimental conditions, such as culture media, likely contributed to the observed differences in antimicrobial susceptibility.

Busscher et al evaluated five bacterial species, including two *S. mutans* strains, in chitosan-treated biofilms using fluorescence microscopy and the LIVE/DEAD BacLight Bacterial Viability Kit.^9^ While chitosan did not detach adhering bacteria, it significantly reduced bacterial viability compared to the buffer control. In the present study, no significant differences in total biofilm biomass were observed between the chitosan and control groups, consistent with Busscher et al’s findings. However, unlike Busscher et al, this study did not observe a reduction in bacterial viability in chitosan-treated groups based on CLSM analysis. This discrepancy may stem from differences in experimental models. Busscher et al focused on planktonic bacteria on pellicle-treated surfaces, whereas this study examined pre-formed *S. mutans* biofilms on hydroxyapatite discs, which are structurally more resilient and may limit chitosan’s activity. These findings suggest that while chitosan is effective against planktonic bacteria, its impact on mature biofilms may be limited.

There is evidence in the literature suggesting that theobromine may have antimicrobial effects against oral bacteria. Lakshmi et al^32^ reported that theobromine toothpaste showed greater antimicrobial activity than fluoride toothpaste, while Demir et al^19^ found similar effects with herbal toothpastes containing theobromine, propolis, and chitosan. However, neither microdilution testing nor CLSM analysis in the present study demonstrated antibacterial effects of theobromine against *S. mutans*. They differed in their overall formulations beyond the inclusion of specific herbal ingredients, which complicates the direct attribution of the observed antibacterial effects to the herbal components alone. Additionally, both studies employed the agar disc diffusion method, which has limited reliability and validity. In contrast, a recent study by Rafiq et al^41^ investigated the antimicrobial activity of a theobromine solution using the broth microdilution method. Their findings, differing from those of the present study, demonstrated that theobromine exhibited significant antimicrobial activity against planktonic *S. mutans* compared to an untreated control group. These discrepancies may be due to differences in experimental conditions and theobromine formulations.

The study evaluated the antibacterial and anti-biofilm efficacy against a single bacterial species, *S. mutans*, including its mono-species biofilm. While *S. mutans* was chosen due to its key role in the caries process, this represents a limitation, as dental caries is a polymicrobial disease influenced by complex microbial interactions within oral biofilms. Another limitation is the relatively small sample size, which may impact the generalizability of the findings. Future studies with larger sample sizes are needed for validation. Although biofilms were developed under identical conditions across all groups, no direct baseline quantification was performed before treatment. Consequently, biofilm analysis was limited to between-group comparisons, preventing an assessment of each compound’s direct impact on biofilm reduction over time. Finally, this study assessed only the short-term remineralisation effects of the tested compounds, limiting the understanding of their long-term efficacy. Future research should explore their remineralisation potential over extended periods to determine sustained effects.

In conclusion, the data from this study show that micro-CT analysis revealed that chitosan and theobromine demonstrated significant remineralisation effects, while propolis had a limited impact. In contrast, antibacterial assessments showed that propolis exhibited notable antimicrobial and anti-biofilm activity, whereas chitosan and theobromine had minimal effects against *S. mutans*. These findings suggest that while chitosan and theobromine may contribute to enamel remineralisation, propolis is more effective in inhibiting bacterial growth. Further research is needed to explore their long-term effects and potential applications in caries prevention strategies.

## CONCLUSION

In conclusion, this study demonstrates that chitosan and theobromine exhibited significant remineralisation effects, as evidenced by micro-CT analysis, whereas propolis had a limited impact. In contrast, antibacterial assessments showed that propolis exhibited notable antimicrobial and anti-biofilm activity, whereas chitosan and theobromine had minimal effects against *S. mutans*. These findings suggest that while chitosan and theobromine may contribute to enamel remineralisation, propolis is more effective in inhibiting bacterial growth. Further research is needed to explore their long-term effects and potential applications in caries prevention strategies.

### Acknowledgements

The present work was supported by the Research Fund of Istanbul University (project number: 34696).

## REFERENCES
